# Editorial: Autonomous robotic systems in aquaculture: research challenges and industry needs

**DOI:** 10.3389/frobt.2025.1740881

**Published:** 2025-11-21

**Authors:** Eleni Kelasidi, Michael Triantafyllou, Sveinung Johan Ohrem

**Affiliations:** 1 Department of Mechanical and Industrial Engineering, NTNU, Trondheim, Norway; 2 Department of Aquaculture Technology, SINTEF Ocean, Trondheim, Norway; 3 Department of Mechanical Engineering, MIT, Cambridge, MA, United States

**Keywords:** aquaculture, robotics, fish-machine interaction, welfare, machine vision and AI methods

## Introduction

This editorial summarizes the contributions to the Research Topic “Autonomous Robotic Systems in Aquaculture: Research Challenges and Industry Needs”, appearing in the Frontiers in Robotics and AI journal.

As the global population increases, the demand for sustainable and efficient food production continues to grow. Aquaculture, the fastest-expanding food sector, now provides a significant portion of global seafood. Despite its importance, it remains one of the most demanding and risk-prone industries, with challenges in Health, Safety, and Environment (HSE), dependence on manual labor, and reliance on human experience. To ensure its sustainable growth, aquaculture must adopt robotics, automation, and artificial intelligence. This Research Topic in Frontiers in Robotics and AI presents advances demonstrating how autonomous robotic systems are reshaping aquaculture by improving safety, efficiency, and animal welfare, guiding the industry toward digital and sustainable operations.

## Robotics and intelligent sensing in aquaculture

The contributions span the aquaculture value chain, addressing robotic design, underwater sensing, navigation, fish–robot interaction, and welfare monitoring. Central to these efforts is developing intelligent systems that operate reliably in complex and dynamic marine environments. By merging multi-sensor perception, machine vision, and adaptive control, these technologies are redefining farm management and advancing the vision of precision aquaculture.

## Experimental platforms and behavioral research

A key innovation is the Cyber-Enhanced Tank (Voskakis et al.), a sophisticated experimental platform combining event cameras, imaging sonars, and optical systems to simulate real aquaculture environments. This system enables non-invasive monitoring of fish behavior under controlled yet realistic conditions. It bridges laboratory research with industrial practice, supporting the design and validation of new robotic and sensing technologies essential for welfare-conscious automation.

## Deep learning and underwater perception

Autonomous operation underwater faces challenges such as low visibility and dynamic conditions. Advances in computer vision and deep learning have enabled robust 3D tracking of fish and environmental features (Føre et al.). Neural models integrated within the Robot Operating System (ROS2) can estimate fish–vehicle distance and behavioral response in real time, allowing robots to adapt behaviorally and minimize stress. This development is a step toward ethical, biologically aware automation in aquaculture.

## Autonomous inspection and maintenance

Inspection and maintenance of underwater infrastructure are vital yet hazardous and costly when performed manually. A vision-based inspection system implemented on the Blueye X3 drone uses real-time image enhancement, AI-based object detection, and visual servoing for autonomous operation (Nguyen et al.). Built on open-source frameworks, it lowers costs while improving safety and data quality. Such technologies exemplify how scalable, affordable robotics can benefit aquaculture and offshore industries alike.

## Fish welfare and environmental sensitivity

Sustainability in aquaculture depends on animal welfare. Studies on Atlantic salmon have shown that certain acoustic frequencies–especially near 400 Hz–trigger avoidance behavior, while light intensity and depth strongly influence stress levels (Zhang et al.). These findings inform robotic system design, ensuring propulsion noise, light, and sensor emissions are configured to reduce stress and maintain welfare standards.

## Mapping, localization, and digital twins

Accurate mapping and localization are fundamental to robotic autonomy. Combining monocular depth prediction with acoustic sensing allows unmanned underwater vehicles (UUVs) to estimate positions and create detailed 3D maps in real time (Job et al.). These capabilities enable precise inspection, continuous monitoring, and digital twin creation of aquaculture sites, paving the way for intelligent and integrated farm management systems.

## Artificial intelligence in ecosystem monitoring

Beyond aquaculture production, AI is also advancing environmental monitoring. The ODYSSEE model, designed for oyster identification, was compared with human experts and non-experts (Campbell et al.). Although AI achieved faster results with slightly lower accuracy, the study underscores its potential for non-invasive biodiversity assessment and habitat conservation. Improved datasets and annotation methods will enhance such applications, enabling scalable, AI-driven ecosystem monitoring.

## Behavioral analytics for welfare and productivity

Computer vision enables real-time monitoring of fish behavior in sea cages. By analyzing correlations between fish activity, depth distribution, and environmental conditions such as temperature, wave motion, and light, researchers identified behavioral adaptations that reduce exposure to stressors (Burke et al.). Continuous monitoring supports adaptive management, offering a foundation for welfare-based automation that improves both productivity and fish health.

## Toward an intelligent and sustainable aquaculture future

Collectively, these studies illustrate how robotics, AI, and sensing technologies are converging to transform aquaculture into a data-driven, efficient, and ethically grounded industry. The combination of perception, control, and autonomy with welfare-focused design represents a major step toward Industry 4.0 for the marine domain.

The innovations summarized here underscore the essential role of technology in ensuring a sustainable future for food production from the sea. From intelligent sensing and mapping systems to welfare-aware robots and digital twins, these developments enable precision aquaculture that balances productivity with environmental responsibility. As the need for sustainable protein grows, robotics and AI are not merely technological tools–they are vital to ensuring that aquaculture continues to feed the world reliably and responsibly.

